# Inherited Renal Tubulopathies—Challenges and Controversies

**DOI:** 10.3390/genes11030277

**Published:** 2020-03-05

**Authors:** Daniela Iancu, Emma Ashton

**Affiliations:** 1 UCL-Centre for Nephrology, Royal Free Campus, University College London, Rowland Hill Street, London NW3 2PF, UK; 2 Rare & Inherited Disease Laboratory, London North Genomic Laboratory Hub, Great Ormond Street Hospital for Children National Health Service Foundation Trust, Levels 4-6 Barclay House 37, Queen Square, London WC1N 3BH, UK; emma.ashton@gosh.nhs.uk

**Keywords:** inherited tubulopathies, next generation sequencing, genetic heterogeneity, variant classification.

## Abstract

Electrolyte homeostasis is maintained by the kidney through a complex transport function mostly performed by specialized proteins distributed along the renal tubules. Pathogenic variants in the genes encoding these proteins impair this function and have consequences on the whole organism. Establishing a genetic diagnosis in patients with renal tubular dysfunction is a challenging task given the genetic and phenotypic heterogeneity, functional characteristics of the genes involved and the number of yet unknown causes. Part of these difficulties can be overcome by gathering large patient cohorts and applying high-throughput sequencing techniques combined with experimental work to prove functional impact. This approach has led to the identification of a number of genes but also generated controversies about proper interpretation of variants. In this article, we will highlight these challenges and controversies.

## 1. Introduction

Mutations in genes that encode transporter proteins in the renal tubule alter kidney capacity to maintain homeostasis and cause diseases recognized under the generic name of inherited tubulopathies. Hereditary tubulopathies constitute a heterogeneous group of rare diseases with a global prevalence that is still difficult to estimate. Although by combining genetic and functional studies, we have reached a better understanding of the mechanisms of these disorders, and the genetic cause for a significant number of affected patients is still unknown. This may derive from the variable clinical picture, overlapping phenotypes, insufficient exploration of the genome, mostly of non-coding areas, or incomplete knowledge about the genes governing renal tubular function. 

Next-generation sequencing (NGS) has become increasingly accessible for both research and clinical practice and brought new challenges into the interpretation process [1,2,3]. Some of the challenges associated with it are derived from the technology itself (a higher number of variants in genes other than the gene of interest, incidental discoveries eliciting ethical issues, hard to sequence genomic regions) [1,4]. Other challenges are related to the limited information available to interpret the variants. Genetic heterogeneity, pleiotropy, variable penetrance, and expressivity, overlapping phenotypes, at least during the early stages, may influence interpretation. Bartter and Gitelman syndromes represent the best examples in this respect [5,6,7]. Occasionally, acquired disorders can mimic genetic tubulopathies (for example, a Gitelman-like syndrome as a rare consequence of Sjögren syndrome) and thus initiate a diagnostic odyssey for the patient. In this article, we aim to highlight the most frequent and recent challenges and controversies raised by the genetic diagnosis of inherited tubulopathies. 

## 2. Exploring the Genetic Causes of Tubulopathies 

The evolution of genetic technologies has facilitated the discovery of a large number of disease genes, initially starting with the identification of a candidate gene based on its linkage with certain markers transmitted with the disease in affected families (positional cloning). Sanger sequencing was introduced in 1977 [8] and has supported gene discovery and diagnosis for more than four decades. Human genes vary in length from several hundreds to a few million base pairs [9], which makes Sanger sequencing a very inefficient method for mutation detection in large genes or sets of genes. It is still used in clinical practice to validate results obtained through faster, high-throughput methods. Next-generation sequencing of whole or targeted exomes or even whole genomes circumvented issues like the partial overlap of certain phenotypes and the genetic heterogeneity [10,11,12]. This progress was accompanied by the discovery of a subset of variants that can be difficult to interpret with current knowledge [13,14,15]. To safeguard the accuracy of genetic diagnosis, guidelines for variant interpretation have been developed and published by the American College of Medical Genetics [16]. These recommendations proved to be very useful in clinical context, when variants in validated genes are investigated. An initial evaluation of how different labs apply these criteria showed substantial disagreement [17], but following subsequent discussions led to an improved consistency [18,19]. However, when new genes or new inheritance patterns are discovered, these guidelines are insufficient. Experimental evidence has shown that the physicochemical nature of an amino acid change, conservation through multiple species, and the rarity of the variant are not always indicative of pathogenicity [20,21,22]. Once large-scale sequencing became available and the results collected into large populational datasets like Exome Aggregation Consortium (ExAc) [23] and Genome Aggregation Database (gnomAD) [24], some of the variants previously considered to be causal for a rare disease have been re-classified as uncertain significance or non-pathogenic [21,25] after being found in large numbers in the control population. 

## 3. Animal Models Do Not Always Reflect Human Disease

One aspect impeding a better understanding of the changes of renal physiology induced by mutations in genes responsible for tubular transport is the inconsistent reproduction of the phenotype in animal models [26]. For example, the mouse expressing *GATM* (Glycine Amidinotransferase) mutants causing AD renal Fanconi syndrome did not show any aminoaciduria or glucosuria [27]. This is also dependent on the type of genetic modification: (a) knock-out mouse reproducing the effect of a loss-of-function variant is the most used model; (b) knock-in mouse obtained by editing the gene to include the precise pathogenic variant identified in patients. A knock-in experiment introducing the missense *WNK4* (WNK Lysine Deficient Protein Kinase 4) mutation p.(Q562E) identified the pathogenic mechanism resulting in pseudohypoaldosteronism type II in humans and a similar phenotype in mice [28]. In the case of *SLC34A1* (Solute Carrier Family 34 Member 1), two mouse models of Slc34a1 deficiency either due to genetic deletion or to the spontaneous occurrence of the compound heterozygous mutations at p.(L499V) and p.(V528M) show no Fanconi syndrome [29,30]. However, a mouse bearing two Slc34a1 missense mutations presented electrolytic abnormalities due to a trafficking defect [30,31]. OCRL (Inositol Polyphosphate-5-Phosphatase) KO mice did not exhibit Lowe syndrome/Dent disease unless Inpp5b (Inositol Polyphosphate-5-Phosphatase B) was deleted in the proximal tubule as well. This is due to the specific redundancy of Ocrl and Inpp5b in mice which required a deletion of either both genes or replacement of Inpp5b with the human gene to obtain similar phenotype to the one observed in Lowe patients [32]. The zebrafish model has impaired endocytosis, but normal cilia function [33]. *SLC26A1* (Solute Carrier Family 26 Member 1) is an anion exchanger with expression in a limited number of tissues (liver, gut, and kidney). The KO mice exhibit hyposulfatemia, hypersulfaturia, calcium oxalate urolithiasis, and nephrocalcinosis [34]. In addition, they have an increased sensitivity to acetaminophen, which is a significant aspect for pharmacogenetics studies in humans [34]. The differences between humans and mice or other animal models challenge our capacity to document the functional effects of genetic variants.

## 4. Large-Scale Sequencing Projects Have Variable Diagnostic Yields

Two large multicentre studies identified a mutation in 70% of the children [7] and about 26% in adults [6] with clinically diagnosed tubulopathies. Another study found a genetic diagnosis in 7% of 235 Pakistani patients diagnosed with nephrolithiasis [35]. Braun et al. found a monogenic cause of nephrolithiasis (NL) or nephrocalcinosis in close to 17% [36] of the patients, in which the recessive mutations were more frequent than the autosomal dominant. This proportion is similar to another study that included both children and adults with NL [37]. Smaller scale projects were dedicated to distal renal tubular acidosis (dRTA) [38], Dent disease [39,40], and Gitelman syndrome [41] providing genetic confirmation of the diagnosis in variable proportions. The high number of cases with an unresolved genetic diagnosis can be explained by the complex nature of tubulopathies, with both genetic and environmental factors contributing to the disease, as well as the limited knowledge about the contribution of other genes, the small number of patients with some very rare conditions and phenotype variability. The number of studied genes differed in each case. An internationally reviewed resource, PanelApp, attempts to gather expert knowledge to support inclusion of new genes and a permanent review of known genes as part of gene panels for molecular diagnosis [42]. The tubulopathy panel contains 57 genes of which 38 are validated with high confidence (https://panelapp.genomicsengland.co.uk/panels/292/; accessed 27/02/2020) according to the number of cases, functional studies supporting causality and other supporting evidence. Two genes, HNF4A (Hepatocyte Nuclear Factor 4 Alpha) and SLC2A2 (Solute Carrier Family 2 Member 2) causing renal Fanconi syndrome 4 and Fanconi-Bickel syndrome, respectively, are not yet classified due to the reduced number of cases published so far. The panel can be exported and used for selecting genes to be analyzed.

## 5. Genetic and Phenotypic Heterogeneity Complicate Genetic Analysis

Genetic heterogeneity and variations in phenotype are probably the biggest challenges in the genetic diagnosis of renal tubulopathies, and this is illustrated by Table 1 containing the genes associated with inherited tubulopathies and the corresponding phenotypes described for each gene.

Fanconi renotubular syndrome (FRTS) is one of the best examples of genetic heterogeneity and phenotype variation. The renal Fanconi syndrome may be inherited or secondary to nephrotoxic substances, autoimmune diseases, or cancer [43]. Four entities have been clustered so far under the name of renal Fanconi syndrome, three autosomal dominant and one autosomal recessive [44,45]. The renal phenotype is the result of impaired reabsorption in the proximal tubule, resulting in loss of water, phosphate, glucose, bicarbonate (HCO3-), uric acid, aminoacids, and low molecular weight proteins [43]. The patients present with polyuria and polydipsia, impaired growth, rickets and osteopenia; in time, they may develop renal insufficiency [43,46]. Three forms (FRTS1, FRTS3, and FRTS4) are validated and follow an autosomal dominant (AD) inheritance pattern while the fourth (FRTS2), which is autosomal recessive, is still debated due to the limited number of cases and insufficient experimental evidence. In addition, a number of other multisystemic, metabolic inherited disorders may associate with renal Fanconi syndrome: cystinosis, galactosemia, hereditary fructose intolerance, tyrosinemia, Wilson disease, and mitochondrial diseases, reviewed in [43,47]. This heterogeneity adds to the fact that sometimes the full clinical picture develops in time, thus delaying appropriate management and increasing the risk of renal failure [48]. Therefore, the early diagnosis is a challenge for the clinician and requires a broad molecular investigation. 

FRTS1 was initially linked to a different location at 15q15.3 by Lichter-Konecki et al. [50], but the gene could be identified only after re-analysis of the data extending the region to include the flanking markers and thus the *GATM* gene [27]. The mutations impair mitochondrial clearance by generating long polymers that prevent mitochondrial fission and degradation [27] in the renal proximal tubules. *GATM* encodes for L-arginine:glycine amidino-transferase, an enzyme whose gene product was originally isolated from pig kidney mitochondria [51]. Interestingly, biallelic nonsense mutations had been described as the cause for cerebral creatine deficiency syndrome 3 (OMIM# 612718) with autosomal recessive inheritance [52,53] and without any documented renal phenotype. 

FRTS3 is caused by monoallelic mutations in the *EHHADH* gene (enoyl-CoA hydratase/3-hydroxyacyl CoA dehydrogenase), an enzyme involved in peroxisomal oxidation of fatty acids [49]. The phenotype had been described in 1955 by Luder at al. [54] and Tolaymat et al. [55] and it is reviewed in [47]. 

FRTS4 [56] has been identified by Hamilton et al.; in patients displaying both features of mature onset diabetes of young (MODY) and proximal renal tubulopathy associating nephrocalcinosis, hypercalciuria and hypermagnesemia, hypocalcemia, and renal impairment. The neonates with this condition have hyperinsulinism and increased birth weight.

More controversies surround the association between mutations in *SLC34A1* and autosomal recessive FRTS, type 2 [45]. A biallelic in-frame duplication of 21 bp found in two children from a consanguineous family leads to complete loss of function due to a defective membrane localisation of the mutated protein [45]. No further cases have been published and thus OMIM considered this gene-phenotype association as provisional, in concordance with PanelApp [42], which maintains this association in the “Red” zone. *SLC34A1* encodes for the NaPiIIa transporter which contributes by about 70–80% of the apical influx of sodium and phosphate, thus being the major effector of phosphate reabsorption in the kidney [57]. Phosphate is mostly reabsorbed in the proximal tubule, and there is no known secretion along the tubule. Reduced systemic phosphate determines adaptive changes with suppression of *FGF23* (Fibroblast Growth Factor 23), increased 1-alpha hydroxylation of vitamin D, and suppressed PTH, mobilisation of calcium and phosphate from bones and increased calcium deposits in kidneys (nephrocalcinosis). Mutations in *SLC34A1* are also causal for AR infantile hypercalcemia [58,59,60], with many cases reported in the literature. Conversely, it was proposed by some [35,61,62,63] and contested by others [64,65,66] that monoallelic mutations in the same gene determine AD hypophosphatemia and nephrocalcinosis, particularly in association with environmental factors. A dominant negative effect of heterozygous NaPiIIa mutations could not be demonstrated and phosphate transport has been mildly affected in some of the experiments [64,66]. In all cases, the proposed disease mechanism is a classical loss-of-function, with mostly point mutations (missense, splicing, nonsense, frameshift) identified. Two mutations, p.(I456N) and p.(R512C), were shown to impair trafficking to the membrane [63]. SLC34A1 deficiency seems to have a higher impact at earlier ages, while in adulthood the phenotype becomes milder [57]. *SLC34A1* mutations have been found in five cases of infantile hypercalcemia of a total of 410 children with tubulopathies underlining the rarity of this condition [7]. A similar study focusing on 1033 adult patients identified only two patients with renal hypophosphatemia and heterozygous variants in *SLC34A1* [6]. It is accepted that SlC34A1 is characterised by an increased number of single allele non-synonymous variants in the general population, with increased predisposition to developing kidney stones [67,68] and chronic kidney disease [69,70]. 

*SLC34A3* (Solute Carrier Family 34 Member 3) is another member of the SLC34 gene family expressed in the proximal tubule which contributes less to phosphate reabsorption and has been associated with AR hypophosphatemic rickets with hypercalciuria (HHRH) [71,72]. In this case, both recessive and dominant inheritance are largely accepted, following the discovery that heterozygotes have been seen in a number of cases with hypercalciuria and kidney stones but without bone disease and with inconsistent hypophosphatemia [73]. One of the mutations, p.(Ser192Leu), is more frequent among Europeans and associated with a less severe renal and osseous phenotype in homozygous form [74] and with increased predisposition to renal calcification (NL/NC) when heterozygous [68,75]. Experimental studies demonstrated a reduced phosphate transport function of the mutant channel in different cellular systems (*Xenopus* oocytes and Human embryonic kidney cells, HEK293) and the high frequency in gnomAD database (99 alleles out of a total of 214,524; 88 of 91,194 alleles in Europeans) interpreted in the context of a milder phenotype [74]. Similar experiments were not able to support any evidence of functional impairment in monoallelic cases [74]. Both transporter proteins may adjust their cell surface expression in accordance with hormonal factors and phosphate intake [76]. Missense mutations can prevent proper localisation of the protein at the membrane [77]. Carriers of some *SLC34A3* mutations may present with hypercalciuria [71], but a functional study of two carriers in a family of a HHRH patient showed no biochemical abnormality [77]. In fact, both *SLC34A1* and *SLC34A3* are known to harbor a large number of monoallelic variants, as shown by control populational databases [57]. 

Another gene involved in phosphate reabsorption is *SLC20A2* (Solute Carrier Family 20 Member 2), but, unlike the *SLC34A1* and *SLC34A3*, it is expressed ubiquitously and the associated phenotype involves brain calcifications but no renal features [78]. *XPR1* is a gene presumed to be expressed in the basolateral membrane, where it might export phosphate into the blood stream [79]. There is no renal disease associated with mutations in this gene, despite the kidney being one of the organs with higher expression; instead, several missense mutations have been reported to cause a form of basal ganglia calcification [80]. Although no pathogenic variants associated with a renal phenotype have been identified in humans, the mouse in which Xpr1 gene had been conditionally inactivated exhibited a clear picture of renal tubular dysfunction [81]. A better understanding of these genes and more detailed experimental studies may shed light on the intricate physiology of the proximal tubule.

## 6. Mutations outside the Known Pattern for the Condition

Variation in some genes like *SLC5A2*, *AQP2*, *KLHL3*, *SLC4A1*, or *SLC2A9* may follow either an autosomal recessive or autosomal dominant pattern, with one of these being the rule while the other is occasionally seen. The disease mechanism might differ between AD and AR even for those cases where the phenotype is similar [82]. This is not unique to tubulopathies and raises a challenge for the clinical interpretation of new variants that are not yet experimentally proven to be pathogenic. Experiments with either heterozygous, homozygous, or compound heterozygous knock-in models may orient the interpretation, and the new genome-editing technologies and organoid models are expected to clarify many of the questions we still have today [83]. In most cases, the disease mechanism is of loss-of-function in both AD and AR cases but also gain-of-function is seen in some of the AD conditions [82]. Thus, autosomal dominant *SLC5A2* is caused by a reduced transport function, to about 70% of the wild-type level [84]. Variants in *KLHL3* cause Pseudohypoaldosteronism type IID in either monoallelic (AD) or biallelic (AR) combinations. The difference is that the variants causing AD disease are clustered at intra- or intermolecular interaction sites, impeding functional interactions [85]. AD NDI-causing *AQP2* mutations are located towards the C-terminal end and exert a dominant-negative effect [86]. The same clustering and disease mechanism is seen in *SLC4A1* [87], while a loss-of-function mechanism has been found for *SLC2A9* [88].

Deep intronic mutations may escape detection, unless suspected and investigated separately or within a more general, whole-genome approach. They have been cited in *SLC34A3* [89,90], or *SLC12A3* [91,92]. Since targeted panel sequencing is the commonest approach, these rare cases might be missed unless supplementary tests are performed. Regularly, the introns are considered the less conserved parts of a gene, where most variations may accumulate without significant consequences, unless they change one of the critical regions, the canonical donor and acceptor sites or the branch site. However, occasionally, some of these variants disrupt regulatory regions or other genes, introduce a new, ectopic splice site, resulting in an insertion of a new sequence (pseudo-exon) that can be in-frame or shift the reading frame and ultimately lead to insertion of a premature stop codon [93]. Deep intronic mutations are reported in more than 77 disease-associated genes [93]. The *SLC5A2* gene encodes for the major sodium-glucose cotransporter in the kidney [94]. Monoallelic and biallelic mutations are responsible for isolated renal glycosuria [95,96,97]. Besides several missense and truncating mutations, an intronic variant consisting of a deletion of 20 nucleotides between −10 and −31, presumably affecting the branching site, has been published [98]. As whole genome sequencing becomes more cost-efficient and broadly used, it is expected that the number of reported mutations in this category will increase.

Occasionally, a tubulopathy gene may be affected as part of a contiguous gene deletion and lead to a syndromic presentation. An example is a deletion comprising *SLC34A1* and *NSD1* determining the association of severe hypophosphatemia to Sotos syndrome [99]. Therefore, once any sign of renal tubular impairment is seen in a patient, it is worth exploring potential additional causes as this can be critical for disease management and genetic counselling in the family.

A recent international survey identified 6 out of 36 patients with no clear causative variant in any of the known dRTA genes as being heterozygous for the variant ATP6V1B1: c.1181G>T, p.(Arg394Gln). Since the second pathogenic variant has not been identified in these cases [100] as well as others [7,101], it has been suggested that this mutation might be an example of autosomal dominant inheritance, but this still needs experimental proof. 

A heterozygous mutation (c.265G>A; p.(A89T)) added glycosuria to the phenotype of an otherwise unrelated disease, juvenile cataract with microcornea, caused by monoallelic *SLC16A12* mutations. Because *SLC16A12* was found to be also expressed in the kidney, the association had been initially reported as a new syndrome [102,103]. Further segregation studies demonstrated that glucosuria was a separate phenotype [104]. Thus, a broader genetic investigation in the case of an unusual presentation of a disorder is more appropriate to clarify the diagnosis and guide management and family counselling as the occurrence of two independent genetic disorders, however exceptional, must not be disregarded.

## 7. Digenic Inheritance

Mutations in more than one gene may exceptionally be associated with an unusual phenotype [105]. With replacement of single gene sequencing by whole exome or whole genome sequencing, it is likely that many more such cases will surface where pathogenic variants are found in two or more genes, suggesting the so-called digenic or oligogenic inheritance. One question that might be asked in such situations is whether this is a true digenic effect [106] or the second gene bears a mutation only by chance. In diseases with variable phenotype like renal tubulopathies, this is more difficult to assess. It is known that some genes are more tolerant to non-synonymous variation [107]. A number of renal transporters can be included in this category: *SLC34A1* is already a famous example, acknowledged by many publications and the allelic frequency in populational databases. 

According to the cases reported so far in tubulopathies and in other conditions, several situations can be recognized: (1) two genes causing similar phenotypes are mutated simultaneously according to the recognised individual pattern and generate a more severe or variable phenotype [108]; (2) two genes from the same pathway and with known functional and/or physical overlap suffer from inactivating mutations with a different phenotype [109]; and (3) variants in two or more genes, inconsistent with the pattern of inheritance characteristic for the disease [110,111]. 

Bartter and Gitelman syndromes are the commonest renal tubulopathies [5,112]. Clinical and biochemical characteristics are detailed here [5]. There are five main forms of Bartter syndrome, each with an independent genetic cause and a subset of characteristic biochemical features [5]. Clinical presentation is not always suggestive and may not be similar in all affected members in a family. Expert consensus guidelines [112] have been drawn up to guide efficient genetic testing in GS patients. One interesting and challenging aspect is the number of digenic inheritance reports following the initial case of Bartter syndrome and deafness caused by inactivating biallelic mutations in both *CLCNKA* and *CLCNKB* [109,113]. The patient presented with a similar phenotype to Bartter syndrome 4A, known to be caused by mutations in *BSND* [114]. More recently, next-generation sequencing results suggested more cases of digenic inheritance in Bartter and Gitelman syndromes, one of which involves monoallelic variants in *CLCNKB* and *SLC12A3* genes [111]. There is no demonstrated dominant negative effect of a heterozygous *CLCNKB* or *SLC12A3* mutation and no experiment has proven that in heterozygous state these variants would have any effect at all on protein stability, trafficking, or transport function. Carrier relatives of patients with either *CLCNKB* or *SLC12A3* associated Gitelman syndrome are healthy. The two chloride channels, CLCNKA and CLCNKB, are known to interact and to be co-expressed in certain parts of the nephron and internal ear. They both require BSND for stability and membrane localisation [115]. In the ascending loop of Henle, *CLCNKA* is less expressed, which means that inactivating mutations in *CLCNKB* result in salt loss and impaired mineral homeostasis as a result of reduced chloride export from the renal tubule cells [116,117]. Through inactivating mutations of both genes, there is a severe loss of saline transport associated with hearing loss due to defective formation of endolymph [113]. This phenotype is similar to that generated by mutations in *BSND*, thus illustrating the interdependence of the three genes [114]. Interestingly, only homozygous inactivating mutations in each of the two chloride channels, *CLCNKA* and *CLCNKB*, have been associated with deafness, perfectly overlapping with the definition of true digenic effects. Digenic inheritance requires a demonstrable interaction between the two genes, a clear consistence across a larger pedigree or multiple pedigrees [105], which could be proven for Bartter syndrome 4B but would be less convincing in other cases. Exome and genome sequencing result in a larger number of potentially damaging variants, which make them more demanding in terms of analysis and interpretation. Similar results have been reported in dRTA [118]. 

A patient with Dent 2 disease was found to have mutations in both *CLCN5* and *OCRL* genes, and an intermediary phenotype between DD and LS [108], while another case with *OCRL* and *INPP5B* variants presented with a Chiari I malformation [119]. 

Given the number of non-synonymous mutation in the genes encoding for transporter proteins, it is very likely that pathogenic mutations can be present independently in more than one gene thus requiring a thorough experimental, genetic, and physiological investigation before supporting a non-canonical inheritance pattern. Interestingly, some of the genes associated with Mendelian diseases are also the site of relatively rare variants that confer increased susceptibility to common forms of renal diseases (nephrolithiasis, chronic kidney disease) [120,121,122].

While the occurrence of damaging variants in two or more genes in one individual is not impossible, we can suggest that these cases must be validated experimentally before being used to manage and counsel patients and families.

## 8. Conclusions

Next-generation sequencing has the potential to speed up gene discovery and thus improve management of patients with inherited tubulopathies through a more precise molecular diagnosis. One effect of the technology can be generation of a large list of variants of unknown significance that can lead to overinterpretation and false diagnostic association, unless stringent criteria are applied to classify and interpret them.

## Figures and Tables

**Table 1:** Genes associated with inherited tubulopathies.

Gene	Alias	Official Name	OMIM	Associated Phenotype	OMIM	Inheritance1
Proximal tubule **						
**GATM**	AGAT	Glycine amidinotransferase	602360	Cerebral creatine deficiency syndrome 3	612718	AR
				Renal Fanconi syndrome and kidney failure		AD
EHHADH	LBFP; LBP	Enoyl-CoA hydratase and 3-hydroxyacyl CoA dehydrogenase	607037	Fanconi renotubular syndrome 3	615605	AD
HNF4A	TCF14, HNF4	Hepatocyte nuclear factor 4-alpha	600281	Fanconi renotubular syndrome 4, with maturity-onset diabetes of the young	616026	AD
				MODY, type I	125850	AD
SLC2A2	GLUT2	Solute carrier family 2 (facilitated glucose transporter), member 2	138160	Fanconi-Bickel syndrome	227810	AR
**SLC22A12**	URAT1, OAT4L	Solute carrier family 22 (urate transporter), member 12	607096	Hypouricemia	220150	AR
**SLC2A9**	GLUT9	Solute carrier family 2 (facilitated glucose transporter), member 9	606142	Hypouricemia, renal, 2	612076	AR, AD
**REN**	RENIN	Renin	179820	Hyperuricemic nephropathy, familial juvenile 2	613092	AD
				Renal tubular dysgenesis	267430	AR
CLCN5 *	CHLORIDE CHANNEL, VOLTAGE-GATED, K2; CLCK2; CLC5	Chloride voltage-gated channel 5	300008	Dent disease	300009	XLR
				Hypophosphatemic rickets	300554	XLR
				Nephrolithiasis, type I	310468	XLR
				Proteinuria, low molecular weight, with hypercalciuric nephrocalcinosis	308990	XLR
OCRL *	OCRL1	OCRL, inositol polyphosphate-5-phosphatase	300535	Dent disease 2	300555	XLR
				Lowe syndrome	309000	XLR
**KCNJ10**	GLIAL INWARDLY RECTIFYING POTASSIUM CHANNEL Kir4.1	Potassium voltage-gated channel subfamily J member 10	602208	Enlarged vestibular aqueduct, digenic	600791	AR
				EAST/SESAME syndrome	612780	AR
**SLC5A2**	SGLT2	Solute carrier family 5 (sodium/glucose cotransporter), member 2	182381	Renal glucosuria	233100	AR, AD
ABCG2	ABCP BCRP MRX	ATP binding cassette subfamily G member 2	603756	[Junior (Jr) blood group system]	614490	
				[Uric acid concentration, serum, QTL1]	138900	?AD
SLC9A3R1 *	NHERF1 EBP50	Solute carrier family 9, MEMBER 3, regulator 1	604990	Nephrolithiasis/osteoporosis, hypophosphatemic, 2	612287	AD
XPR1	SLC53A1, SYG1	Xenotropic and polytropic retrovirus receptor	605237	Basal ganglia calcification, idiopathic, 6	616413	AD
**FAH**	FUMARYLACETOACETASE	Fumarylacetoacetate hydrolase	613871	Tyrosinemia, type I	276700	AR
**CTNS**	CYSTINOSIN	Cystinosin, lysosomal cystine transporter	6060272	Cystinosis, atypical nephropathic	219800	AR
				Cystinosis, late-onset juvenile or adolescent nephropathic	219900	AR
				Cystinosis, nephropathic	219800	AR
				Cystinosis, ocular nonnephropathic	219750	AR
Thick ascending loop of Henle (TAL) and Distal Convoluted Tubule (DCT) **						
**SLC12A1**	NKCC2	Solute carrier family 12 (sodium/potassium/chloride transporter), member 1	600839	Bartter syndrome, type 1	601678	AR
**KCNJ1**	ROMK; ROMK1, KIR1.1	Potassium voltage-gated channel subfamily J member 1	600359	Bartter syndrome, type 2	241200	AR
**CLCNKB**	CLCKB	Chloride voltage-gated channel Kb	602023	Bartter syndrome, type 3	607364	AR
				Bartter syndrome, type 4b, digenic	613090	DR
**BSND**	BARTTIN	Barttin CLCNK type accessory beta subunit	606412	Bartter syndrome, type 4a	602522	AR
CLCNKA	CLCK1	Chloride voltage-gated channel Ka	602024	Bartter syndrome, type 4b, digenic	613090	DR
**MAGED2**	MELANOMA ANTIGEN, FAMILY D, 2;	MAGE family member D2	300470	Bartter syndrome, type 5, antenatal, transient	300971	XLR
**SLC12A3**	NCC, NCCT	Solute carrier family 12 (sodium/chloride transporter), member 3	600968	Gitelman syndrome	263800	AR
**TRPM6**	CHAK2;	Transient receptor potential cation channel subfamily M member 6	607009	Hypomagnesemia 1, intestinal	602014	AR
FXYD2	ATP1G1	FXYD domain containing ion transport regulator 2	601814	Hypomagnesemia 2, renal	154020	AD
**CLDN16**	PCLN1	Claudin 16	603959	Hypomagnesemia 3, renal	248250	AR
EGF	UROGASTRONE; URG	Epidermal growth factor	131530	Hypomagnesemia 4, renal	611718	AR
**CLDN19**	CLAUDIN 19	Claudin 19	610036	Hypomagnesemia 5, renal, with ocular involvement	248190	AR
**ATP1A1**		ATPase Na+/K+ transporting subunit alpha 1	182310	Charcot-Marie-Tooth disease, axonal, type 2DD	618036	AD
				Hypomagnesemia, seizures, and mental retardation 2	618314	AD
**HNF1B**	HNF2	HNF1 homeobox B	189907	Diabetes mellitus, noninsulin-dependent	125853	AD
				Renal cysts and diabetes syndrome	137920	AD
KCNA1	MK1, MOUSE, HOMOLOG OF KV1.1	Potassium voltage-gated channel subfamily A member 1	176260	Episodic ataxia/myokymia syndrome	160120	AD
				Autosomal dominant hypomagnesemia	No MIM	AD
**NR3C2**	MLR; MCR; MR ALDOSTERONE RECEPTOR	Nuclear receptor subfamily 3 group C member 2	600983	Hypertension, early-onset, autosomal dominant, with exacerbation in pregnancy	605115	AD (gain of function p.Ser810Leu)
				Pseudohypoaldosteronism type I, autosomal dominant	177735	AD
**WNK4**	PRKWNK4	WNK lysine deficient protein kinase 4	601844	Pseudohypoaldosteronism, type IIB	614491	AD
WNK1	PSK PRKWNK1 _ KDP KIAA0344	WNK lysine deficient protein kinase 1	605232	Neuropathy, hereditary sensory and autonomic, type II	201300	AR
				Pseudohypoaldosteronism, type IIC	614492	AD
**KLHL3**	KELCH-LIKE 3	Kelch like family member 3	605775	Pseudohypoaldosteronism, type IID	614495	AD, AR
**CASR**	PARATHYROID CA(2+)-SENSING RECEPTOR 1; PCAR1	Calcium sensing receptor	601199	Hyperparathyroidism, neonatal	239200	AD, AR
				Hypocalcemia, autosomal dominant	601198	AD
				Hypocalcemia, autosomal dominant, with Bartter syndrome	601198	AD
				Hypocalciuric hypercalcemia, type I	145980	AD
				{Epilepsy idiopathic generalized, susceptibility to, 8}	612899	
**GNA11**	GUANINE NUCLEOTIDE-BINDING PROTEIN, ALPHA-11	G protein subunit alpha 11	139313	Hypocalcemia, autosomal dominant 2	615361	AD
				Hypocalciuric hypercalcemia, type II	145981	AD
**AP2S1**	CLAPS2, AP17	Adaptor related protein complex 2 sigma 1 subunit	602242	Familial hypocalciuric hypercalcemia type III	600740	AD
**CYP24A1**	CYTOCHROME P450, SUBFAMILY XXIV;	Cytochrome P450 family 24 subfamily A member 1	126065	Hypercalcemia, infantile, 1	143880	AR
SLC34A1 *	NaPiIIa	Solute carrier family 34 (type II sodium/phosphate cotransporter), member 1	182309	?Fanconi renotubular syndrome 2	613388	AR
				Hypercalcemia, infantile, 2	616963	AR
				Nephrolithiasis/osteoporosis, hypophosphatemic, 1	612286	AD
SLC34A3 *	NaPiIIc	Solute Carrier Family 34 Member 3	609826	Hypophosphatemic rickets with hypercalciuria	241530	AR
CLDN10	OSPL CPETRL3	Claudin 10	617579	HELIX syndrome (anhidrosis, heat intolerance, renal sodium chloride wasting, mild renal failure)	617671	AR
**UMOD**	Tamm-Horsfall Glycoprotein; THP; THGP	Uromodulin	191845	Glomerulocystic kidney disease with hyperuricemia and isosthenuria	609886	AD
				Hyperuricemic nephropathy, familial juvenile 1	162000	AD
				Medullary cystic kidney disease 2	603860	AD
Collecting Duct (CD) **						
**ATP6V0A4**	ATP6N1B ATP6N2 VPP2	ATPase H+ transporting V0 subunit a4	605239	Distal Renal Tubular Acidosis, Recessive	602722	AR
**ATP6V1B1**	ATP6B1 VPP3	ATPase H+ transporting V1 subunit B1	192132	Renal tubular acidosis with deafness	267300	AR
**SLC4A1**	BND3, AE1	Solute carrier family 4 (anion exchanger), member 1	109270	Cryohydrocytosis	185020	AD
				Ovalocytosis, SA type	166900	AD
				Renal tubular acidosis, distal, AD	179800	AD
				Renal tubular acidosis, distal, AR	611590	AR
				Spherocytosis, type 4	612653	AD
**SLC4A4**	NBC1	Solute carrier family 4 (sodium bicarbonate cotransporter), member 4	603345	Renal tubular acidosis, proximal, with ocular abnormalities	604278	AR
**CA2**		Carbonic anhydrase 2	611492	Osteopetrosis, autosomal recessive 3, with renal tubular acidosis	259730	AR
**AQP2**	AQUAPORIN-CD	Aquaporin 2	107777	Nephrogenic diabetes insipidus	125800	AD AR
**AVPR2**	ADHRV2R	Arginine vasopressin receptor 2	300538	Diabetes insipidus, nephrogenic	304800	XLR
				Nephrogenic syndrome of inappropriate antidiuresis	300539	XLR
**SCNN1A**	SCNEA; SCNN1	Sodium channel epithelial 1 alpha subunit	600228	?Liddle syndrome 3	618126	AD
				Bronchiectasis with or without elevated sweat chloride 2	613021	AD
				Pseudohypoaldosteronism, type I	234350	AR
**SCNN1B**	SCNEB	Sodium channel epithelial 1 beta subunit	600760	Bronchiectasis with or without elevated sweat chloride 1	211400	AD
				Liddle syndrome 1	177200	AD
				Pseudohypoaldosteronism, type I	264350	AR
**SCNN1G**	SCNEG	Sodium channel epithelial 1 gamma subunit	600761	Bronchiectasis with or without elevated sweat chloride 3	613071	AD
				Liddle syndrome 2	618114	AD
				Pseudohypoaldosteronism, type I	264350	AR
**CUL3**		Cullin 3	603136	Pseudohypoaldosteronism, type IIE	614496	AD
GNAS	GNAS1	GNAS complex locus	139320	McCune-Albright syndrome, somatic,	174800	mosaic
				Osseous heteroplasia, progressive	166350	AD
				Pseudohypoparathyroidism Ia/Ib/Ic	103580/603233/612462	AD
				Pseudopseudohypoparathyroidism	612463	AD
SLC20A2	PIT2	Solute carrier family 20 (phosphate transporter), member 2	158378	Basal ganglia calcification, idiopathic, 1	213600	AD
				Mutations found in renal patients but unclear phenotype		
WDR72	WD REPEAT-CONTAINING PROTEIN 72	WD repeat domain 72	613214	Amelogenesis imperfecta, type IIA3	613211	AR

AR: autosomal recessive; AD: autosomal dominant; XLR: X-linked recessive; * these genes are on the ‘Red’ list as they are also part of other panels. ** the segments are indicated based on their clinical significance and not necessarily as the only regions where the corresponding genes are expressed. The genes shown in bold are included in the ‘Green’ List of the Renal Tubulopathy Panel. Note: the rest of the genes are classified as ‘Amber’ or ‘Red’ based on a reduced number of reported cases or insufficient experimental evidence. The list corresponds to the PanelApp version 2.3 (https://panelapp.genomicsengland.co.uk/panels/292/; accessed 27/02/2020). Two AD variants of renal Fanconi syndromes, FRTS1 and FRTS3, are linked to genes involved in tubular cell metabolism, thus underlying the importance of energy supply for the proper functioning of transport mechanisms [27,47,49].
